# Extracting Behaviorally Relevant Traits from Natural Stimuli: Benefits of Combinatorial Representations at the Accessory Olfactory Bulb

**DOI:** 10.1371/journal.pcbi.1004798

**Published:** 2016-03-03

**Authors:** Anat Kahan, Yoram Ben-Shaul

**Affiliations:** Department of Medical Neurobiology, Hebrew University Medical School, Jerusalem, Israel; University of Tübingen and Max Planck Institute for Biologial Cybernetics, GERMANY

## Abstract

For many animals, chemosensation is essential for guiding social behavior. However, because multiple factors can modulate levels of individual chemical cues, deriving information about other individuals via natural chemical stimuli involves considerable challenges. How social information is extracted despite these sources of variability is poorly understood. The vomeronasal system provides an excellent opportunity to study this topic due to its role in detecting socially relevant traits. Here, we focus on two such traits: a female mouse’s strain and reproductive state. In particular, we measure stimulus-induced neuronal activity in the accessory olfactory bulb (AOB) in response to various dilutions of urine, vaginal secretions, and saliva, from estrus and non-estrus female mice from two different strains. We first show that all tested secretions provide information about a female’s receptivity and genotype. Next, we investigate how these traits can be decoded from neuronal activity despite multiple sources of variability. We show that individual neurons are limited in their capacity to allow trait classification across multiple sources of variability. However, simple linear classifiers sampling neuronal activity from small neuronal ensembles can provide a substantial improvement over that attained with individual units. Furthermore, we show that some traits are more efficiently detected than others, and that particular secretions may be optimized for conveying information about specific traits. Across all tested stimulus sources, discrimination between strains is more accurate than discrimination of receptivity, and detection of receptivity is more accurate with vaginal secretions than with urine. Our findings highlight the challenges of chemosensory processing of natural stimuli, and suggest that downstream readout stages decode multiple behaviorally relevant traits by sampling information from distinct but overlapping populations of AOB neurons.

## Author Summary

Across the animal kingdom, chemical senses play a central role in guiding social behaviors by conveying information about particular behaviorally relevant traits. However, decoding these traits from profiles of chemical cues is challenging since cue levels are modulated by multiple factors. Here, we investigate how the mouse vomeronasal system, a chemosensory system important for processing social information, detects behaviorally relevant traits from natural stimuli. We focus on detection of a female’s genetic background (a model for individuality) and estrus-state (a measure of sexual receptivity) by neurons in the first vomeronasal brain relay, the accessory olfactory bulb (AOB). We show that information about both genetic background and receptivity can be obtained from various stimulus sources: urine, vaginal secretions, and saliva. Importantly, while individual AOB neurons can only provide limited decoding ability of these traits, simple networks sampling AOB neuronal ensembles provide considerable improvement. Our analyses highlight an overlooked challenge associated with chemosensory processing and suggest how it can be overcome by downstream neurons that read information from multiple AOB neurons.

## Introduction

Social animals are extremely adept at extracting information about conspecifics and many species rely on chemosensory cues to achieve this goal [1–3]. Yet, deriving information about specific traits pertaining to other individuals can be complicated by several factors [4]. One factor is physical variability, for example due to stimulus source dilution. Namely, if a given trait is associated with a particular level of some compound, dilution or concentration of the stimulus source could confound correct trait detection [4, 5]. Another factor is the influence of multiple ecologically relevant traits on the levels of any one type of molecule. For example, analysis of mouse urinary compound levels as a function of genetic background and reproductive state indicates that both factors modulate the levels of all tested compounds. In some cases, even the *direction of change* as a function of reproductive state varies with the genetic background [6]. A third factor involves the stimulus source identity. Many animals, including rodents [7, 8], investigate multiple body regions of conspecifics and thus inevitably sample different secretions. Compound concentrations likely differ across secretions and interpretation of their content must thus account for the secretion sampled. Given these sources of variability, reliable detection of any trait becomes a significant computational challenge.

Here, we explore the neural representation of genetic background (a model for identity) and female estrus-stage (a measure of receptivity), two traits that are critical for a male mouse to guide reproductive behavior [2, 9, 10]. We study neuronal responses at the level of the accessory olfactory bulb (AOB), the first brain stage receiving vomeronasal inputs [11]. The vomeronasal system (VNS) is ideal for this study as its role in the detection of both of these traits is well established [9, 10, 12–16]. In particular, the two donor strains used here (BALB/c and C57BL/6), are clearly distinguishable by mice in the context of the vomeronasal-system mediated pregnancy block effect (The Bruce effect) [17, 18]. Furthermore, physiological recordings reveal that responses of vomeronasal sensory neurons, and of AOB neurons, are modulated by both the reproductive state [10, 12, 19–22], and the strain [7, 19, 23–25].

An important element in this study is the parallel investigation of multiple chemosensory stimulus sources: urine, saliva and vaginal secretions. While urine has been extensively studied as a chemosensory stimulus source in rodents [2, 22, 25–27], saliva and vaginal secretions have received much less attention. Here we show for the first time that neurons in the AOB respond to all these secretions in a strain and reproductive-state specific manner. However, we find a major difference between sensitivity to a certain trait and the ability to reliably detect it in the presence of multiple sources of variability. Thus, while many individual neurons can provide some information about these traits, any one neuron in isolation is generally insufficient to provide invariant information about them. We show, however, that integration of information across multiple neurons considerably improves trait detection, and that the detection does not rely on a small number of specialist neurons. Our study highlights the complexities of extracting socially relevant information from chemosensory cues, yet also suggests that relatively simple networks can overcome these challenges.

## Results

### AOB responses to multiple stimulus sources reflect the donor’s strain and estrus state

**Fig 1:**
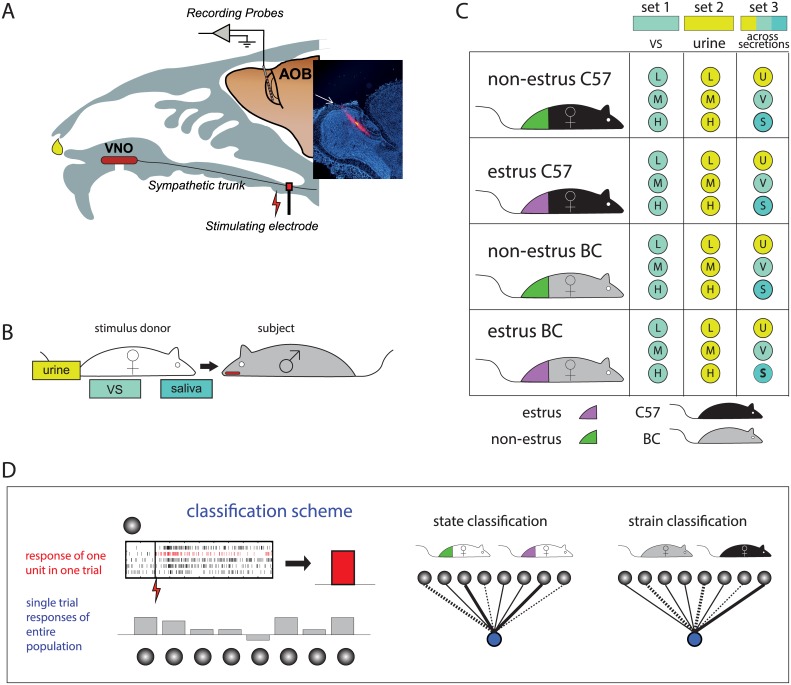
Experimental approach. **A**. The experimental preparation. Stimuli are presented to the VNO through the nostril, and then the sympathetic nerve trunk is stimulated with a cuff electrode to induce VNO suction. Extracellular multi-site probes (32 channels) were targeted to the external cell layer of the AOB. Inset shows a 20μm sagittal section including the main and accessory olfactory bulbs. One tract made by a probe dipped in DiI (red) prior to insertion can be seen. Blue: DAPI nuclear stain. **B**. Illustration of stimulus sources (secretions) used. **C.** Datasets used in this study. L,M,H represent stimuli of low, medium and high concentrations (for vaginal secretions: L = 9x dilution, M = 3x, H = 1x, for urine: L = 300x, M = 100x, H = 33x). In stimulus set 3, stimuli were not diluted following collection. **D.** Outline of the classification approach. Single-trial responses (see rasters for five trials, one of which is highlighted in red) for each unit were defined as the mean firing rate change following stimulus presentation (indicated by bar). Response vectors of all units are then used to train and test specific classifiers. The cartoon networks on the right show classifiers with hypothetical downstream units (blue) receiving inputs from AOB units (gray). The cartoons illustrate that different classifiers may assign different weights to each of the units.

**Fig 2:**
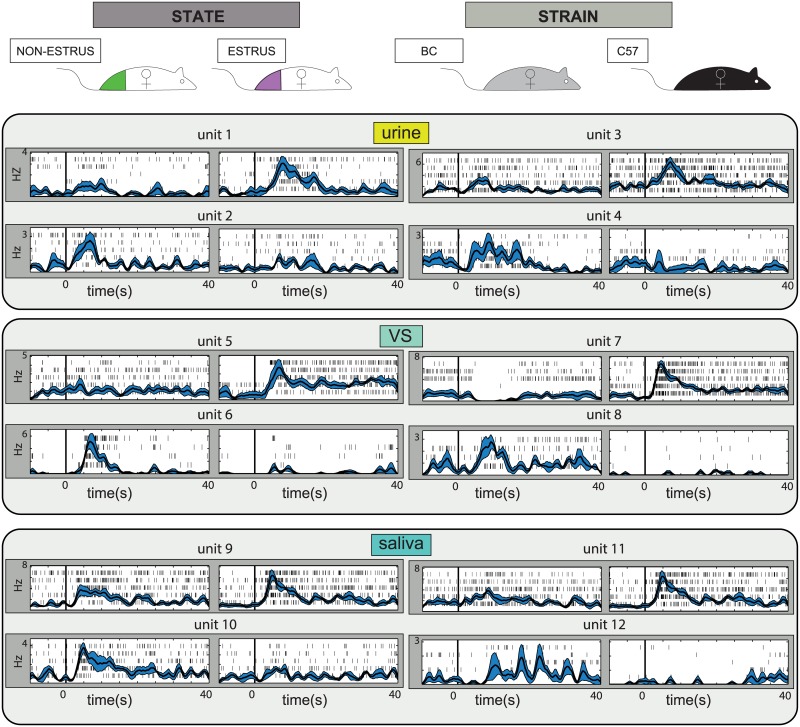
AOB neurons respond to multiple secretions and are modulated by both the strain and the reproductive state of the stimulus donor. Each of the twelve panels show two stimulus- induced responses of one unit. The mean firing rate is superimposed on single trial raster displays following stimulus delivery (time 0 corresponds to sympathetic trunk stimulation). Blue shadings represent the standard error of the mean firing rate. Two units are shown for each secretion/trait combination to illustrate that rates can increase (and sometimes decrease) in response to stimuli associated with each of the traits. VS: vaginal secretions.

We initially tested whether AOB responses to urine, saliva and vaginal secretions can convey the strain and the state of the stimulus donor. Recordings were performed in anesthetized BALB/c males (BC), using multi-electrode arrays. The electrodes were targeted to the AOB external cell layer (Fig 1A) which contains the cell bodies of AOB projection neurons, known as AOB mitral/tufted cells [11]. Stimuli were collected from estrus and non-estrus adult BC and C57BL/6 (C57) female mice (Fig 1B and 1C). Fig 2 shows examples of twelve different units, each of which is modulated by the stimulus donor’s reproductive state (left), or its strain (right), for one of the three secretions.

### Classification approach

While clearly selective to the females’ reproductive-state or strain, the examples shown in Fig 2 are limited to a single pairwise comparison, in which only one trait is varied with all other factors remaining identical. To investigate how invariant representations of strain and state can be attained, we assume the position of a decoder with access to AOB neuronal activity. Unless specified otherwise, we use a simple linear classifier—a perceptron [28]—whose goal is to discriminate among trait values. As inputs, the classifier accepts single-trial responses, defined as averaged firing rate changes calculated during the 40 s period following stimulus presentation. The rate change is defined with respect to the 30 s period preceding vomeronasal stimulation. Fig 1D shows one such single trial (highlighted by the red rasters), used to derive a single value characterizing the neuron’s response (indicated by bar on right side). The broad 40 s time-window was chosen to account for the slow and temporally variable responses of AOB neurons (see Fig 2). A classifier generally receives inputs from multiple neurons (Fig 1D), with single-trial responses sampled independently from each of the neurons. For each classification, we train the classifier with one set of single-trial responses (training set, see Materials and Methods), obtaining a set of weights and a bias term. Following training, classifier performance is tested on a different set of single-trial responses (test set). We first train the classifier with all units in the dataset, and then sequentially remove the unit assigned with the smallest absolute weight. Unless indicated otherwise, classification results denote an average over 10 training cycles. Repeated training cycles were conducted to account for randomness in the training and testing steps.

### Testing the effect of physical variability: Deriving reproductive state information from various dilutions of vaginal secretions

Vaginal secretions are likely to play an important chemosensory role during anogenital investigation of females [3, 29]. However, it is not known what type of behaviorally relevant information can be derived from this stimulus by the VNS. We thus first focused on decoding reproductive state from vaginal secretions. Our dataset includes 92 AOB units (38 single-units, 54 multi-units, 8 sites from 5 mice, Fig 1C set 1, see S1 Table) that responded to at least one of the 12 stimuli at the 0.05 significance level. The mode, median and mean number of significant responses per unit is 1, 3, and 4.0, respectively (See S2 Fig, which also shows that the fraction of responsive units increases with stimulus concentration). Recording site locations of the 92 responding units spanned the entire anterior-posterior aspect of the AOB, indicating that AOB neurons receiving both basal and apical vomeronasal sensory inputs [11, 30] were sampled (S1 Fig).

**Fig 3:**
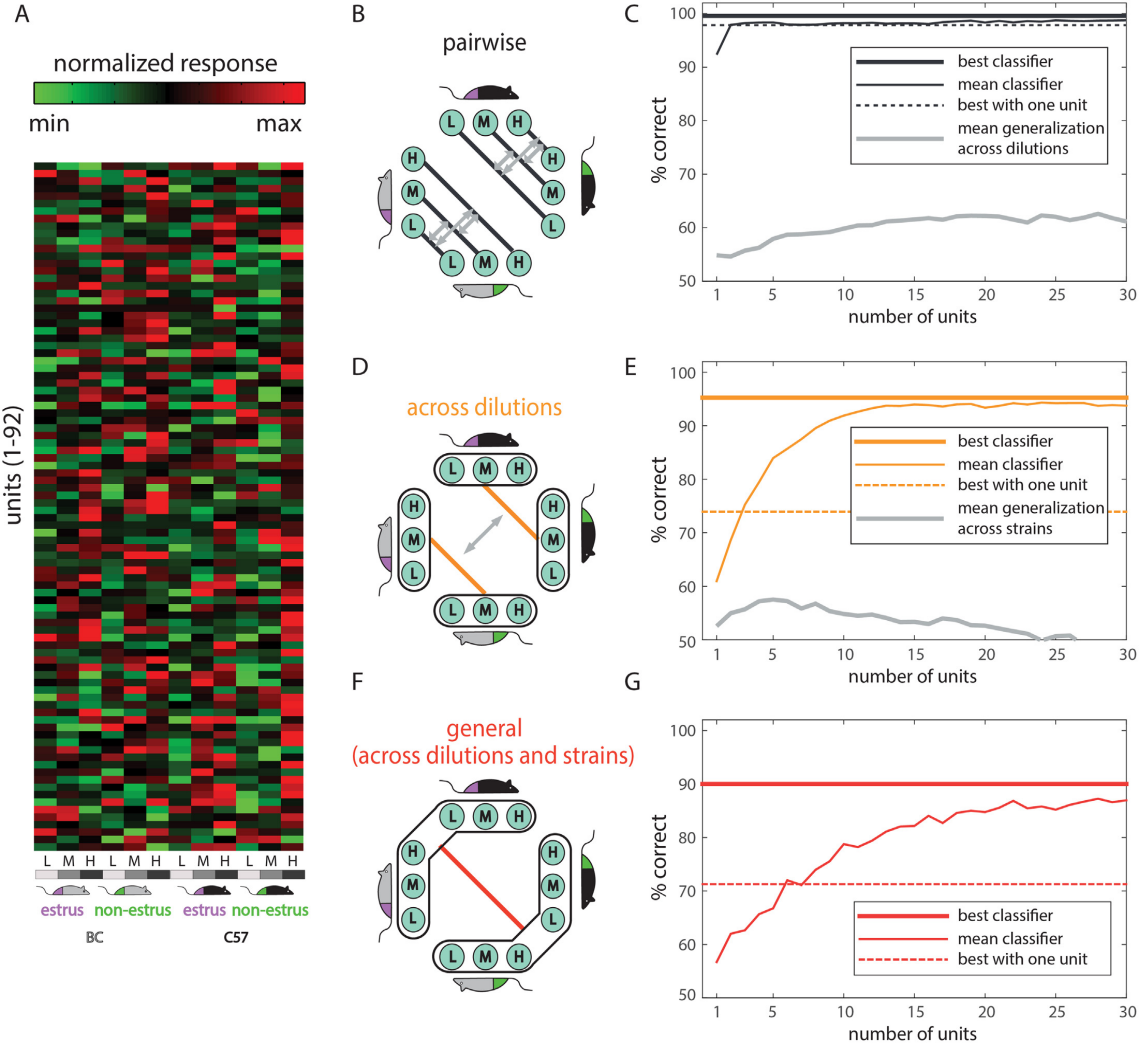
Classification of reproductive state from vaginal secretions. **A.** Response matrix showing normalized responses of AOB units responsive to at least one vaginal secretion stimulus (n = 92 units, stimulus set 1). Each row shows the normalized responses of one unit to the twelve stimuli indicated at the bottom. **B-C.** Pairwise discriminations. The six different comparisons are indicated by black lines in B, and the average classifier performance on those as a function of the number of units is shown in C. The gray double-headed arrows in B represent reciprocal tests of generalization, in which one classifier is tested with the data used to train the other, and vice versa. Average performance on the generalization tests is indicated by the gray traces in C. Plots are truncated at 30 units because further inclusion of units did not significantly improve performance in this analysis. **D-E:** Schematic and performance on dilution invariant classifications (orange), and generalization across strains (gray). **F-G**: Classification of reproductive state across dilutions *and* strains. Panels C, E, and G also show the best perceptron performance as a thick solid line, and the best single unit performance as a broken line. The “best classifier”, and “best single neuron” lines represent averages of the best performance obtained with each individual classifier within a category (there are six classifiers in the simple pairwise category in panels B-C, and two classifiers in the across-dilution category in panels D-E).

The normalized response profiles of all units included in this analysis are shown in Fig 3A. We began with the simplest discrimination, involving two reproductive states, while all other stimulus properties (dilution and strain) remain identical. In our dataset, with two strains and three dilutions, this amounts to six *simple* discriminations (indicated by the black lines in Fig 3B). The classification performance as a function of the number of units, averaged across all 6 pairwise comparisons, is shown in Fig 3C (mean classifier, solid black lines). The traces reveal that even with individual units, all six discriminations can be made with a very high success rate.

We next asked if a classifier trained on one dilution performs well on stimuli at a different dilution. For example, how does a classifier trained to distinguish estrus from non-estrus C57 vaginal secretions at 3x dilution perform on a 1x dilution of the same stimuli? The gray double-headed arrows in Fig 3B indicate the twelve possible tests of generalization. The arrows are double-headed to indicate that two reciprocal tests of generalization can be made with each pair of dilutions. The resulting classifications, whose average is shown by the gray line in Fig 3C, reveal that generalization across dilutions is only slightly better than chance. In this context, it is interesting to note that different urine concentrations were shown to convey different signals, via activation of distinct populations of vomeronasal sensory neurons [22]. See S3A–S3F Fig, for a more detailed description of classification results, and S4 Fig for a detailed examination of generalization across specific dilutions.

Do these observation imply that a classifier cannot perform well across different dilutions of a stimulus? To answer this, we next trained a classifier to discriminate estrus state across *all* vaginal secretion dilutions (separately for each strain), as illustrated by the orange lines in Fig 3D. Fig 3E shows that robust classification is indeed possible with a simple linear classifier, even across a range of dilutions. Yet, multiple neurons are needed to achieve maximal performance. Using the neuron removal approach, we observe that roughly 10 units allow classification with a success rate of about 95%. These analyses indicate that classification across dilutions requires explicit training and that polling multiple units provides a substantial benefit.

### Exploring the effect of intrinsic variability: Deriving reproductive state information from different strains

We next address the effect of changing one trait on the ability to discriminate another. Specifically, we test how the two dilution-invariant classifiers, each of which was trained on one strain, perform on stimuli from the other strain. These comparisons are indicated by the double-headed arrow in Fig 3D. The averaged classification performance is shown in gray in Fig 3E. Regardless of the number of units used for classification, generalization across strains yields near chance performance, revealing that the effect of one trait on another is indeed significant. Finally, we trained a classifier to discriminate reproductive state across *both* dilutions and strains (Fig 3F). Although the classifier’s performance plateaued at a lower level, and required more units than the less general classifiers, it clearly yielded above-chance performance (Fig 3G).

The unit removal approach often underestimates the performance of the best individual unit. We therefore also include this value, for comparison, in Fig 3C, 3E and 3G as a broken line (when multiple classifiers are present, the line represents their average). The thick solid line in each of these panels shows the maximal perceptron performance. This value represents the best classification performance over the 10 repeated training and classification cycles, with all units considered. Thus, comparison of the thick solid line with the dotted line in each panel shows the benefit of decoding with multiple units over that possible with any individual unit. While this advantage is minor for the simple classifications (Fig 3C), it is substantial for the more general classifications (Fig 3E and 3G). See S11 Fig for additional analyses showing the limited decoding capacity of individual units.

### Different secretions are optimized to convey information about particular traits

Our analysis thus far was based on one stimulus source (vaginal secretions) and one type of discrimination (reproductive state). We next expand the analysis to another discrimination (strain) and to another secretion (urine). The comparison across discriminations and secretions reveals some general principles but also some notable differences.

The urine dataset comprises 51 units (25 single units, 26 multi-units) that responded to at least one of the 12 urine stimuli (Fig 1C, set 2). Detailed descriptions of the number of units per session and recording site locations, spanning the entire anterior-posterior aspect of the AOB, are given in S1 Table and S1 Fig. For the 51 responding units, the mode, median and mean number of significant responses per unit was 1, 3, and 3.4, respectively (see S2 Fig, which also shows that the number of responsive units increases with stimulus concentration).

**Fig 4:**
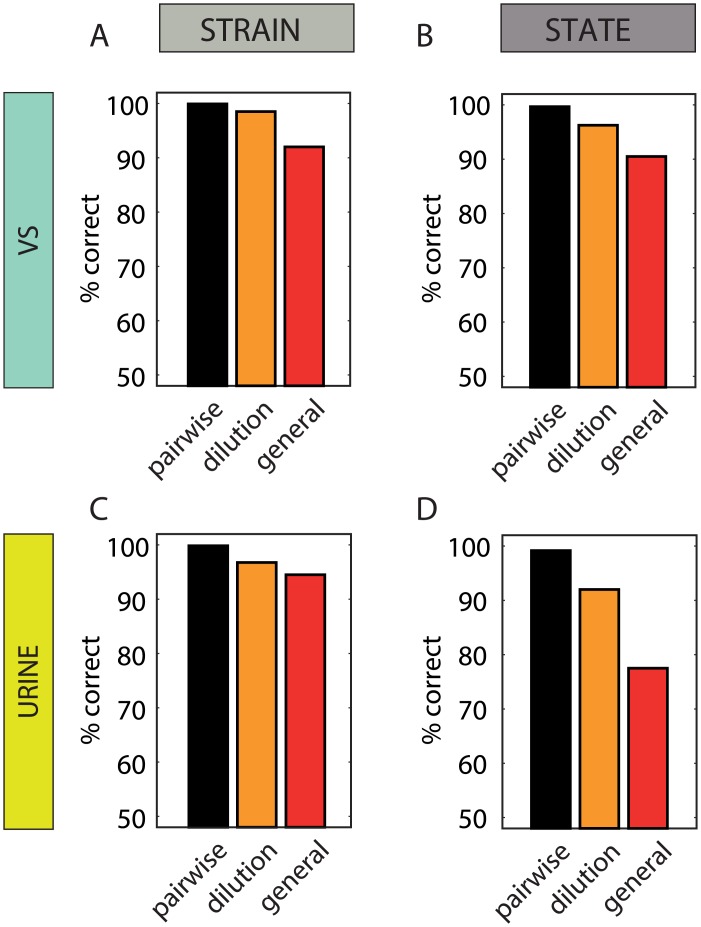
Comparison of state and strain classifications with vaginal secretions and urine. **A.** Classification of strain using vaginal secretions (stimulus set 1). **B.** Classification of state using vaginal secretions. **C.** Classification of strain using urine (stimulus set 2). **D.** Classification of state using urine. Each bar represents the performance of one type of classification with 50 units (the best value over the 10 repeated training cycles is shown). When multiple classifications of a given type exist (i.e. six pairwise and two dilution invariant classifications), the mean values are shown.

Complete descriptions for VS and urine classifications are shown in S3A–S3L Fig. Fig 4 summarizes the classification performance to facilitate comparison across the various cases. To fairly compare the VS and urine datasets (the latter containing fewer units), we consider performance with 50 units. The similarities are highlighted by noting that for both secretions and for both traits, discriminations involving more sources of variability result in poorer performance (panels 4A-D). Comparison of the left-side panels (4A, C) and the right-side panels (4B, D), reveals that for both secretions, strain discriminations are more successful than state discriminations. Comparing the upper and lower panels, particularly for the more difficult, general classifications (red bars), shows that while strain discriminations are marginally better with urine stimuli (4C vs. 4A), state discriminations are considerably more successful with vaginal secretions (4B vs. 4D). Thus, in the context of vomeronasal chemosensation, different secretions seem optimized for conveying distinct socially relevant traits.

### Generalization across stimulus sources

Many mammals, and mice in particular, investigate each other in a manner that inevitably samples multiple stimulus sources [7]. From a decoder’s perspective, the challenge is that levels of different cues, and their dependence on particular traits, could vary across secretions. This consideration is particularly relevant in the context of slow vomeronasal sampling, which can lead to mixing of stimuli from different sources within the vomeronasal organ [31, 32].

In the foregoing analyses, each classifier was trained and tested on a single stimulus source. To investigate how classification criteria based on one secretion apply to others, we recorded responses of AOB neurons to urine, vaginal secretions, and saliva (Fig 1C, set 3). The dataset contains 164 units (101 single-units, 63 multi-units) that responded to at least one of the 12 stimuli. Detailed descriptions of the number of units recorded per session and recording site locations, spanning the entire anterior-posterior aspect, are shown in S1 Table and S1 Fig. For the 164 responding units, the mode, median and mean number of significant responses across units was 1, 2, and 2.6, respectively (see also S2 Fig). Note that in this stimulus set, due to the small volumes of salivary samples, stimuli from four to six females were pooled (see Materials and Methods). This has the potential effect of reducing variability and to some extent simplifying the classification problem.

**Fig 5:**
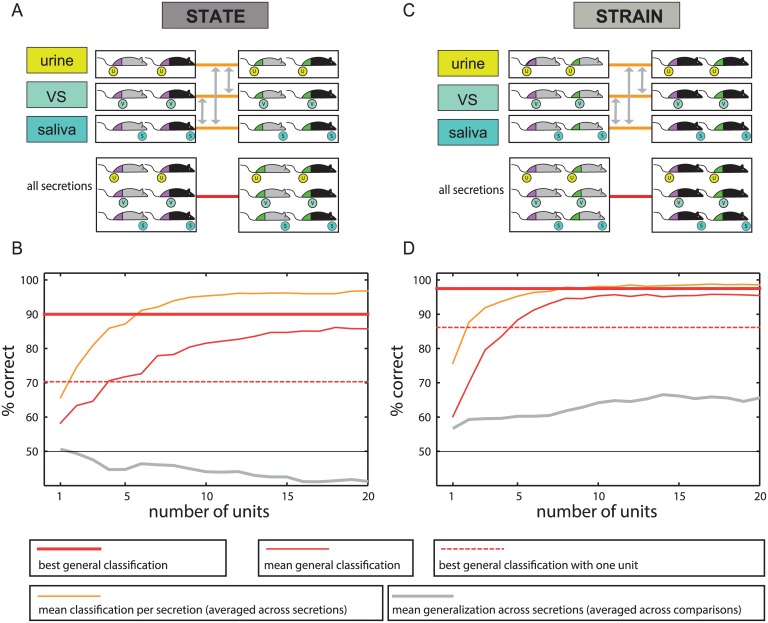
Classification across distinct stimulus sources (stimulus set 3). **A**: Schematic of classifications. Orange lines indicate state classifications for a given stimulus source. Gray arrows indicate tests of classifier generalization across stimuli. The red line indicates general classification across all secretions. **B**: Performance on reproductive-state classifications. The colors correspond to the lines in A. Panels **C** and **D** are similar to A and B, but for strain discriminations. The legend indicates the meaning of the different lines.

We first trained classifiers for each secretion separately. We began with reproductive-state classifiers that generalize across strains, and strain classifiers that generalize across reproductive states (indicated by the orange lines in Fig 5A and 5C). In these experiments, each stimulus is presented at one dilution, and thus dilution variability does not play a factor. The results of these classifications, shown by the orange lines in Fig 5B and 5D, reveal that in addition to urine and vaginal secretions, salivary cues can also provide information about strain and reproductive-state. Complete classification results for this dataset are given in S3 Fig, panels M-R.

Next, we applied classifiers trained on one secretion to the other two (gray double-headed arrows in 5A, C). The analysis shows that application to other secretions results in chance level performance for state discrimination (Fig 5B, gray traces) and slightly above-chance performance for strain discriminations (Fig 5D). These results suggest that distinct chemical cues provide information about behaviorally relevant traits across the different stimulus sources. The modest above-chance generalization performance observed with strain classifications across different secretions indicates that some units do respond to strain dependent cues that are common across secretions.

Finally, we trained classifiers across secretions, to investigate whether a single classifier (red lines in Fig 5A and 5C) can detect particular traits regardless of the stimulus source. Using the unit removal procedure, we observe that about 15 AOB units suffice for approximately 85% correct classification. Best performance with all units, or one unit, is 90% and 70%, respectively. Performance on strain discriminations is higher, with 10 units sufficient to classify strain with a ~95% success rate. Best performance with all units, or one unit, is 98% and 86%, respectively. As with the previous classifications, polling multiple units provides a considerable advantage over individual units. Notably, the ability of one individual unit to provide 86% correct strain discriminations suggests that some neurons are sensitive to strain dependent cues that are common to the three secretions.

### Strain is represented more prominently than reproductive state across the AOB population

**Fig 6:**
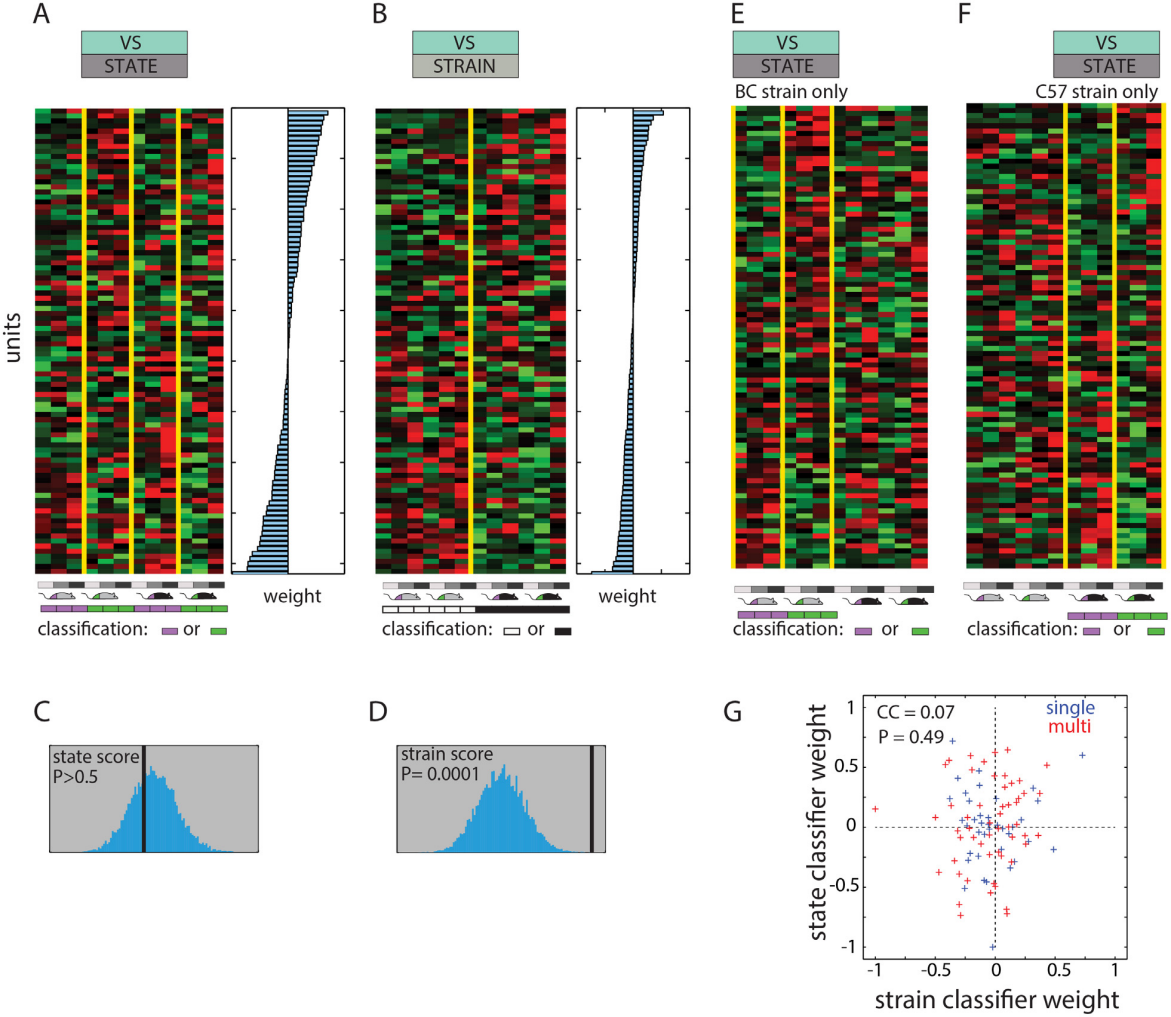
Analysis of classifier weights. **A.** Same data as in Fig 3A, sorted according to the general state classifier weights, which are indicated on the right by blue bars. **B.** Like A, but unit ordering and weights are for the general strain classifier. **C** and **D**, bootstrap analysis of the reproductive-state and strain dimensions in the vaginal secretions dataset. The index corresponding to each dimension is indicated by the black line, while the distribution of this value in shuffled data is shown in blue. Only the strain dimension is represented more prominently than expected by chance. **E.** Same data with units arranged according to the weights assigned by the state classifier for the BC strain. The colored bars below indicate the columns that are used for training and testing the classifier (other columns are ignored during training of this classifier). **F**. Like E, but units are sorted according to the weights assigned by the state classifier for the C57 strain. **G.** Correlation between (normalized) weights for the general vaginal secretion state and strain classifiers (weights shown in panels 6A and B). Single units are indicated in blue, multi-units are shown in red.

To gain insight about how social information is decoded from AOB population activity, we investigated the influence (classifier weights) assigned to each of the units. Fig 6A and 6B show the vaginal secretion response dataset (data shown in Fig 3A) with the rows sorted according to the values assigned by the general reproductive-state and strain classifiers, respectively. As expected, this ordering shows that the linear classifier assigns more weight to units with response profiles that reflect the detected traits. Yet, while unit reordering highlights each of the two traits, it does not reveal if the observed patterns are represented more than expected by chance. To address this, we defined an index that quantifies the impact of the strain and state dimensions on the dataset (see Materials and Methods). Comparison of the index to its bootstrapped distribution under the null hypothesis, reveals that while the strain dimension is significantly represented (Fig 6D), the reproductive-state dimension is not (Fig 6C). The same is true for responses to urine (strain p-value = 0.0035, reproductive-state p-value = 0.55). This observation indicates why strain discriminations are achieved with higher success than state discriminations.

### Generalized classifications are difficult because individual AOB neurons can be modulated by more than one factor

Fig 6E and 6F show the units ordered according to the weights assigned by the reproductive-state classifiers, specifically for the BC and C57 strains (i.e. classifiers indicated by orange lines in Fig 3D). Inspection of the columns representing stimuli not used for training the classifier (i.e. the six rightmost columns in Fig 6E and the six leftmost columns in Fig 6F) reveals that reproductive state selective responses for one strain are not necessarily associated with similar selectivity for the other. Indeed, examination of matrices in Fig 6A and 6B indicates that even units assigned with the highest absolute weights are influenced by the strain and the dilution. This explains why generalization of reproductive state classifiers across strains is not efficient unless training is done *explicitly* with stimuli from both strains. The same observations apply with regard to urine stimuli and for strain discriminations across both states, yet the confounding effect of strain on reproductive state discrimination is more dominant than *vice versa*. As shown in S4 Fig, similar considerations apply to the difficulty of generalizing across concentrations. Generally, we find no systematic relationship between the number of units used and the ability of the classifier to generalize across other instances (S3 Fig). The ability of a classifier to generalize largely depends on the response profiles of the individual units that contribute to it. Specifically, generalization across particular dimensions will be successful if the influential units happen to display invariant responses along these dimensions. To ensure generalization along any given dimension, classifiers must be trained with stimuli that vary along that particular dimension.

### Classifiers do not depend on a small number of highly informative units

**Fig 7:**
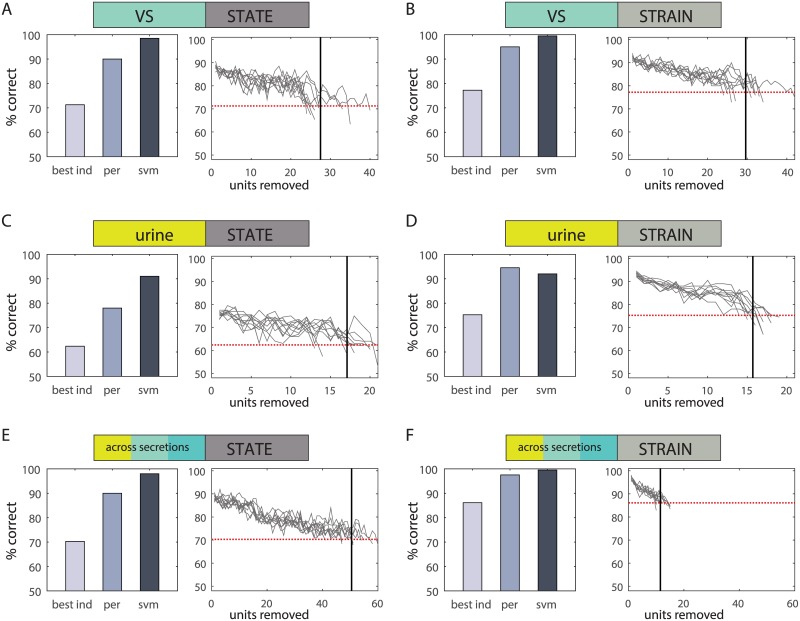
Comparison of classifiers and effects of unit removal. Comparison of the performance of the best individual unit, best perceptron classifier, and best SVM classifier (left in each panel), and an analysis of the effects of unit removal on perceptron performance (right panel). Each panel shows results of 10 repeated unit removal cycles. In each stage of each cycle, the one unit with the highest absolute weight was removed. The dotted red line indicates the performance of the best individual unit. The vertical black line indicates the 10 cycle average of the number of units removed before perceptron performance drops below that obtained with the best individual unit. Values are given in the text. **A.** Reproductive state classifications with vaginal secretions. **B.** Strain classifications with vaginal secretions. **C.** Reproductive state classifications with urine. **D.** Strain classifications with urine. **E.** Reproductive state classifications across secretions. **F.** Strain classifications across secretions.

Across all secretions and traits, our analysis has shown that consideration of multiple units provides better classification performance than is available with any one unit. This is expected, as the entire population of units also includes the “best” unit. This last consideration raises the possibility that the success of classifiers with many units heavily depends on a small number of key units. To address this possibility, we revisited the classification analysis, but instead of removing the unit with the *least* effect (i.e. smallest absolute weight) as done above we instead removed at each stage, the unit with the *highest* absolute weight. For each classifier, the process was repeated until the classifier’s performance decreased below that possible with the best individual unit (Fig 7). The average number of units (across 10 repeated training cycles) that can be removed without impairing classification below the best one-unit performance are: 27.5 (VS state classification), 29.6 (VS strain), 17.1 (urine state), 15.7 (urine strain), 50.5 (across secretions, state) and 11.5 (across secretions, strain). In five out of six cases, about one third of *the most influential* units can be removed while still maintaining performance above that possible with the best individual unit.

S5 Fig illustrates the results of classification with units that were simultaneously recorded in a single session. As expected, classifier performance is substantially reduced in comparison to that obtained with all units available (S3 Fig). Nevertheless, the main conclusions of our foregoing analyses are reproduced even in smaller samples collected in individual sessions.

S6–S8 Figs show an analysis of the correlations among the response profiles of the most influential units for each of the general classifiers. The analysis shows that while there are some prominent response patterns among the influential units, the responses of most unit pairs exhibit low correlations. Specifically, of all pairwise correlations among the top 20 units within each classifier, the large majority are smaller than 0.5. (% correlations below 0.5: VS state: 86.3%; VS strain: 83.7%; urine state: 89.5%; urine strain: 86.3%; across secretions state: 88.4%; across secretions strain: 93.7%). This analysis thus indicates that to achieve optimal performance, classifiers tend to sample units with a variety of response profiles.

Finally, application of Principal Component Analysis (PCA) to our dataset revealed only weak correlations between the dominant Principal Components and the dimensions analyzed here (S9 Fig). This result is consistent with the idea that neurons in our sample do not represent a homogenous population with a one stereotypic response profile.

### Non-linear classifiers yield better classification performance

Our choice of the perceptron model was motivated by its ability to indicate the contribution of individual units to the classification. Furthermore, in principle, it can be easily implemented by neurons receiving direct excitatory and (indirect) inhibitory input from AOB neurons. However, we are *not* suggesting that the actual downstream readout of AOB activity involves a perceptron-like classifier. Indeed, reliable trait detection may be improved by multiple processing stages realizing non-linear computations. To illustrate this, we also calculated the ability of a more powerful non-linear classifier, a support vector machine (SVM), to discriminate among traits. Each panel in Fig 7 compares the best performance obtained with an SVM to that of the best perceptron classifier. The comparison clearly shows that SVMs provide significantly improved performance, particularly for the more challenging classifications. In one case (Fig 7D), perceptron performance is slightly better. This appears surprising because an SVM classifier can implement any decision rule that a perceptron can. The explanation for the reduced performance observed in this case is that the SVM likely over-fits the training dataset.

Comparison of SVM and perceptron performance as a function of the number of units (S10 Fig) shows that the SVM advantage is more prominent when more neurons are used. For small ensembles, the differences between the two classifiers are marginal. Overall, this analysis highlights once more the advantage of polling multiple units, and shows that non-linear readout of neuronal activity can yield improved classification performance.

### Individual units can participate in distinct classifications

**Fig 8:**
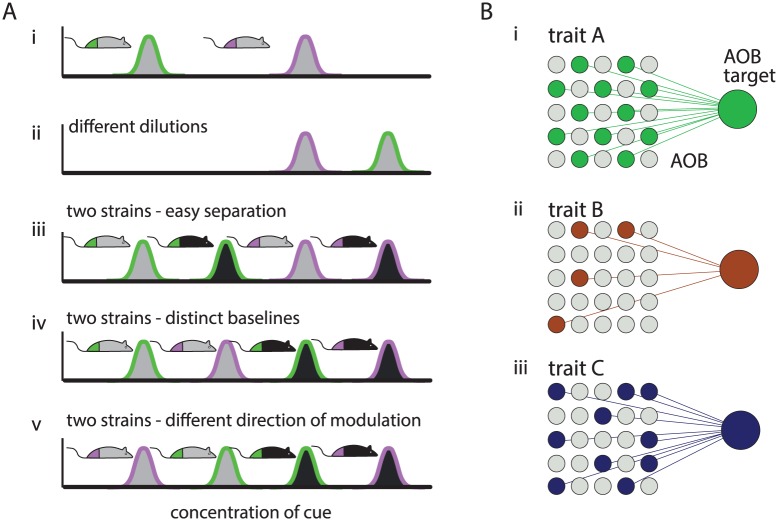
Models of challenges and of solutions for trait detection. **A.** Illustration of the challenges in distinguishing among traits (reproductive state) via levels of a single compound. Each trace corresponds to one strain-state combination. The border and face colors indicate reproductive-state and strain, respectively. In each panel, the horizontal and vertical axes represent compound concentration and probability for that concentration, respectively. i: simple scenario, distinct and non-overlapping distributions of the hypothetical compound in estrus (violet) vs. non-estrus (green) mice. ii: effect of varying dilutions. Here, the non-estrus sample is concentrated, so that compound concentrations are higher than in the estrus stimulus. iii-iv: effect of different baseline levels of the cue in different strains. iii: Separation of estrus and non-estrus is still easy regardless of strain. iv: non-estrus levels of one strain are higher than estrus levels of the other, complicating discrimination. v: strain dependent direction of change. Here, compound levels increase during estrus in one strain, but decrease during estrus in the other. Examples are loosely based on actual data from urinary cues reported in [6]. **B.** Schematic illustrating the idea that a given unit may participate in multiple networks, each associated with detection of one specific trait.

The observation that individual neurons can be modulated by more than one trait implies that each could contribute to the detection of multiple traits. To test whether this is the case, we compared the weights assigned by the reproductive-state and the strain classifiers. Fig 6G shows that across the population of units, the weights for state and strain classifiers for the VS dataset (Fig 6A and 6B) are clearly not correlated. More generally, over 10 classification cycles, the average correlation coefficient between weights for the two distinct classifiers is 0.02±0.03 (mean±SD). For comparison, the average correlation coefficient over all 10 repeated training cycles of the state classifier is 0.99±0.003. Similar results were observed with the other datasets (S2 Table). These results suggest a model according to which individual units participate in multiple distinct and overlapping networks, each associated with classifying a particular trait (Fig 8B).

## Discussion

In this study, we focused on the mouse AOB to study how socially relevant information can be reliably decoded from natural stimuli. In principle, detection of any trait via chemosensation requires a reliable readout of chemical cues that are correlated with the trait. Fig 8A illustrates a very simple (hypothetical) scenario related to the traits addressed here, in which the level of one compound is indicative of estrus state. The example illustrates how factors such as dilution, other traits, or the stimulus source, could modulate compound levels in a manner that confounds discrimination [33]. Even under this simple scenario, trait detection is not a trivial challenge. While there are known cases in which individual molecules elicit well-defined behavioral responses [34–37], encoding even a relatively robust trait such as sex, seems to involve more than a single molecule [38]. More generally, behaviorally relevant information is conveyed by levels of multiple compounds and the relationships between them [2, 4, 20, 25]. These non-trivial relationships between particular traits and levels of chemical cues imply that trait representation in the AOB can be complex.

In one scenario, akin to a labelled line model, the activity of one particular “expert” AOB neuron could provide an unambiguous report of the presence of a particular trait. Under the opposite scenario, the activity of many, potentially all, AOB neurons must be sampled. To provide a definitive answer to this question one would have to test responses of *all* neurons to an enormous set of chemosensory stimuli varying along multiple dimensions, including: various traits of the stimulus donor (e.g. genetic makeup, sex, age, physiological status), the stimulus source-secretion, and physical properties (e.g. dilution, freshness). In attempt to record many neurons with high temporal resolution and fidelity, we used multisite extracellular recording probes in the AOB. Because of technical limitations on practical dataset sizes, we could only explore a limited stimulus subspace in each single experiment (Fig 1C). Specifically, we tested the responses to urine, saliva, and vaginal secretions of females from different strains and reproductive states. We have shown, for the first time, that AOB neurons have the capacity to respond to all these stimulus sources in a strain and reproductive-state specific manner. Furthermore, compared to urine, vaginal secretions are optimized to convey reproductive state information. Linking these results with behavioral analyses, we note that in hamsters, flank marks are used to detect individuality, whereas estrus state is sensed via vaginal secretions [8]. Adding to previous studies [8, 39, 40], our findings stress the importance of attending to diverse secretions in the context of mammalian chemosensory communication.

Based on the responses to these stimuli, we studied how information about a female mouse’s strain and reproductive state can be decoded from the activity of AOB neurons, despite these sources of variability. While some individual units do allow reliable trait discrimination within our (limited) stimulus set, most are significantly restricted in their ability to provide reliable information across multiple sources of variability. However, a significant gain in classification performance is obtained by considering multiple units. In this respect, our work is in good agreement with a recent study, which demonstrated that deriving sex and strain information from urine also requires consideration of multiple neurons [23].

Our analyses indicate that generalized discriminations require explicit training with stimuli spanning multiple dimensions of variability. The challenge is similar to that of object recognition. For example, viewing an unfamiliar face once as a static image is generally not sufficient to allow identification across a range of contexts (e.g. different moods or ages of the person, as well as viewing angles and lighting conditions). The ability to reliably identify a face under those contexts requires accumulated exposure that provides sampling under diverse conditions. Here, we observed that strain discriminations generally required fewer units and were performed with higher success than reproductive-state discriminations. Indeed, our previous analysis has shown that in the AOB, sex is represented more prominently than strain [24]. Generally, prominently represented traits may be more immune to variability, explaining why the confounding effect of strain on state discrimination is larger than the reciprocal effect. Similarly, the confounding effect of stimulus dilution depends on its magnitude relative to that associated with different trait values [4], suggesting why dilution had a minor confounding effect on sex and strain discriminations in the AOB [23].

One may argue that “expert” neurons for trait detection do exist for *all* behaviorally relevant discriminations, but that our limited sample simply failed to identify them. This possibility cannot be entirely ruled out. However, even if such “experts” were found for each discrimination considered here, there is no guarantee that they would also provide reliable classification across further sources of variability. Our present study suggests an alternative scenario, in which discrimination is achieved by polling a limited number of distinct units, none of which must be an “expert”. The various response profiles associated with AOB neurons provide a rich substrate to realize a large number of discriminations, including of traits not strongly represented by neuronal activity. This was exactly the case for reproductive state discriminations in our experiments (Fig 6C). More generally, when traits are strongly represented in the dataset, a small number of units may suffice to yield reliable discriminations (Fig 8Bii), and complex decoding mechanisms might not be required. Thus, instead of a dichotomy between labelled lines and combinatorial codes, we suggest that decoding distinct traits may require different population sizes, depending in part on how prominently these traits are represented by individual neurons.

While the perceptron model provides a clear analogy to a downstream neuron, we do not claim that it actually represents the algorithm used by the brain. Indeed, more powerful non-linear classifiers provide better classification (Fig 7), especially when a larger neuronal population is considered (S10 Fig). Note also that we quantified neuronal responses as the mean firing rate changes within a long time window, a choice motivated by the relatively long and variable response patterns of AOB units [41]. A more refined examination of individual units’ rate modulations and spike timing relative to stimulus delivery might lead to improved decoding ability. In particular, temporal response profiles may play an important role during bouts of natural investigation, when the vomeronasal pump is likely activated repeatedly [7, 31]. The dynamics of active sensing could thus substantially affect summation of responses, and hence the ability to discriminate between, or to generalize across, stimuli. Finally, we note that examination of the relationships between individual units’ spike times, and of correlated rate modulations among different units could also lead to improved discriminations. With all these considerations in mind, understanding how AOB activity is indeed read by specific downstream stages remains an important question for future research.

## Materials and Methods

### Subjects

For recordings, adult sexually unexperienced BALB/C (BC) male mice were purchased from Harlan Laboratories (Israel). All experiments were performed in compliance with the Hebrew University Animal Care and Use Committee. Stimuli were collected from adult sexually unexperienced female mice of the BC and C57BL/6 (C57) strains (Harlan Laboratories, Israel).

### Stimuli

For urine collection, mice were gently held over a plastic sheet until they urinated. The urine was transferred to a plastic tube with a micropipette and then flash-frozen in liquid nitrogen and subsequently stored in -80°C. Vaginal secretions were collected by flushing the vaginal region with 30 μl of ringer’s solution. 20 μl were immediately frozen and stored at -80°C for stimulus presentation. The remaining volume was smeared on a glass slide for determination of estrus state. For saliva collection, isoproterenol hydrochloride (0.2mg/100g) and pilocarpine (0.05mg/100g) were injected I.P. to increase salivation [42]. Saliva was then collected from the oral cavity using a micropipette and immediately frozen in liquid nitrogen and stored at -80°C. All stimulus dilutions were made with ringer’s solution. Stimulus collection was performed 3–5 times per week, usually after 14:00 (non-reversed light cycle, light on: 7:00–19:00). The estrus stage was determined by examining vaginal secretions smeared on glass slides, dried, and stained with cresyl violet. Slides were examined under a light microscope and the estrus cycle stage determined by cellular morphology [43, 44]. Stages were classified as either estrus/proestrus (designated as estrus), or meta- or diestrus (designated as non-estrus). For the urine and vaginal secretion datasets (sets 1 and 2 in Fig 1C), stimuli for a given strain were obtained from one individual (with stimuli collected during different stages of the cycle). Dilutions used were 9x (L, low), 3x (M, medium), 1x (H, high) for vaginal secretions, and 300x (L), 100x (M) and 33x (H), for urine. For comparison of the three different secretions (set 3 in Fig 1C), stimuli comprised a mixture from 4–6 females. In most cases, stimuli for each session were collected from the same females. For comparison of different stimulus sources, we used undiluted samples, as they are thus sampled during natural investigation. With vaginal secretions, this was not possible since they were collected via flushing. Responses to undiluted urine were clearly stimulus specific, and thus did not represent non-specific activation due to urinary potassium S12A–S12D Fig. Unlike in the MOS [45], where airflow alone can induce activity changes, in the VNS, delivery of ringer’s solution alone does not elicit a response [46]. As examples, S12E and S12F Fig shows recordings of two units, demonstrating a null response to Ringers solution. Because the number of stimuli in our dataset was a limiting factor, we did not use an explicit negative control stimulus here.

### Electrophysiology

Experimental procedures were described previously [24], and are reproduced briefly here, with the differences noted. Mice were anesthetized with 100mg/kg ketamine and 10mg/kg xylazine, a tracheotomy was performed with a polyethylene tube to allow breathing during flushing, and a cuff electrode was placed around the sympathetic nerve trunk with the carotid serving as a scaffold. Incisions were closed with Vetbond (3M) glue and the mouse was placed in a custom-built stereotaxic apparatus where anesthesia was maintained throughout the entire experiment with 0.5–1% isoflurane in oxygen. A craniotomy was made immediately rostral to the rhinal sinus, the dura was removed around the penetration site, and electrodes were advanced into the AOB at an angle of ~30° with an electronic micromanipulator (MP-285; Sutter instruments, Novato, CA). All recordings were made with 32 channel probes with 8 channels on each of 4 shanks (NeuroNexus Technologies, Ann Arbor, Michigan). Before recordings, electrodes were dipped in fluorescent dye (DiI, Invitrogen, Carlsbad, CA) to allow subsequent confirmation of electrode placement within the AOB external cell layer, which contains the mitral-tufted cells [11]. In each session, stimuli were typically presented 5 times in a pseudorandom order. In a minority of sessions, only 4 repeats were possible. Mean number of repeats across all experiments: 4.8. In each presentation, 2 μl of stimulus was applied directly into the nostril. After a delay of 20 s, a square-wave stimulation train (duration: 1.6 s, current: ±120 μA, frequency: 30 Hz), was delivered through the sympathetic nerve cuff electrode to induce VNO pumping and stimulus entry to the VNO lumen. Following a second delay of 40 s, the nasal cavity and VNO were flushed with 1–2 ml of ringer’s solution which flowed from the nostril, into the nasal cavity, and sucked out from the nasopalatine duct via a solenoid-controlled suction tube. The cleansing procedure was 50 s long and included sympathetic trunk stimulation to facilitate stimulus elimination from the VNO lumen. Neuronal data was sampled at 25 kHz using an RZ2 processor, PZ2 preamplifier, and two RA16CH head-stage amplifiers (TDT, Alachua, FL). Signals were band-pass filtered (300–5000 Hz) and custom MATLAB (Mathworks, Natick, MA) programs were used to extract spike waveforms. Spikes were sorted automatically according to their projections on two principal components on 8 channels of each shank using KlustaKwik [47] and then manually verified and adjusted using the Klusters program [48]. Spike clusters were evaluated by consideration of their spike shapes, projections on principal component space (calculated for each session individually) and autocorrelation functions. A spike cluster was defined as a *single unit* if it had a distinct spike shape and was fully separated from both the origin (noise) and other clusters along at least one principal component projection, and if its inter-spike interval histogram demonstrated a clear trough around time 0 (of at least 10 ms). Clusters comprising more than one single unit were designated as multi-units. Thus, using the present definitions, multi-units could represent the activity of as few as 2 units, or more. Throughout this manuscript, we used both single and multi-unit activity with the aim of increasing the probability of finding individual units conveying robust information. Note that one of our key conclusions is that response profiles of individual units are not sufficiently general, and this cannot be a result of confounding multiple units together. Comparison of classifier weights assigned to single and multi-units (see Fig 6G) revealed no systematic relationship between unit type and the magnitude of classifier weight.

### Data analysis

All data analyses and visualizations were performed using either custom or standard MATLAB code. The response of a unit to a given stimulus was defined as the average firing rate change over a 40 s window following sympathetic nerve stimulation (change measured compared to the 30 s period preceding VNO activation). Response significance of a given unit to a given stimulus was determined by comparing its spiking rate distribution following all repeats to the baseline firing frequency distribution during the 10 s period prior to stimulus application. Response significance (of a particular unit to a given stimulus) was determined by a non-parametric ANOVA comparing the set of post-stimulation rates to the set of preceding baseline rates (preceding rates were pooled across all stimuli).

#### Classification analyses

The perceptron and the learning rules are described in http://www.mathworks.com/help/nnet/ug/perceptron-neural-networks.html. Here, we used the *perceptron* function in MATLAB (neural networks toolbox, R2014a, http://www.mathworks.com/help/nnet/ref/perceptron.html) to create a perceptron network. Default parameters were used except that the *trainr* function, which selects training samples randomly, was used. A maximum of 100 training epochs was applied.

All training and testing procedures were performed on single trial population vectors including all single and multi-units tested with a given stimulus set. Each response vector was generated by randomly selecting one single-trial response independently from each of the units. With a dataset of 50 units and 5 repeats, this represents an enormous number of distinct combinations. Classifiers were first trained on 100 samples (training set), and then tested on an independently generated test set of 100 trials (test set). The process of training modifies the weights and the threshold (bias) to maximize classification performance on the training dataset. For each discrimination, the performance of the classifier is quantified as a function of the population size by sequentially and repeatedly removing the least influential unit (i.e. the one with the lowest absolute weight) from the previous stage, and applying the training and testing procedures on the remaining units. Due to the slight non-deterministic nature of the training, each classification cycle (including training and subsequent testing) was performed 10 times. The performance of classification as a function of population size was obtained by averaging the mean performance over 10 repeats. This sequential unit removal approach does not always converge to the one unit that, considered alone, provides the best performance. Therefore, in all our classifications, we also show the best performance obtained across all individual units considered separately. For the analysis in Fig 7, at each stage, the unit with the *highest* absolute weight was removed, and the procedure was repeated 3 times for each classifier. Support vector machine classifiers were created and trained with the MATLAB *svmtrain* function (statistics toolbox, R2014a) with a quadratic kernel function. See supporting information files (S1 Data) for classifier codes and for the data arrays used for classification.

For significance analysis of trait representation we defined an index to characterize the extent to which different dimensions are represented in the response dataset. For example, to obtain the representation of the *strain* dimension for a unit *i*, we subtract the sum of the responses of that unit to all stimuli from one strain from the sum of the responses to all stimuli from the other strain. e.g.:

$${\text{I}}_{\text{strain,}i}=\left|\sum_{s\;\in \;strain\;1}R_{i,s}-\sum_{s\;\in \;strain\;2}R_{i,s}\right|$$

Here, *R*_*i*,*s*_ is the normalized response of neuron I to stimulus S, such that the maximal response has a value of 1 and the minimal response has a value of -1. If the magnitude of the responses is identical for both strains (on average), then the index will sum to zero. This value is calculated for each unit and then averaged across all units to obtain a measure of the representation of that trait across the population. The statistical significance of this index is obtained by comparing it to a bootstrapped distribution generated by shuffling the dataset 10,000 times and calculating this index for each shuffle. During shuffling, response magnitudes were randomly permuted for each unit across stimuli. This eliminates any dependency of response magnitudes on specific traits, while preserving the statistics of response magnitudes. Finally, the value of the averaged index of the non-shuffled data is compared to its distribution in the shuffled dataset, and the probability of obtaining by chance a value at least as large as that observed is noted. Similarly, the state specific index is derived by summing all responses to non-estrus stimuli and subtracting them from the sum of responses to all estrus stimuli.

Principal component analysis was performed with the MATLAB PCA function.

## Supporting Information

S1 Table List of number of units sampled in each session. Each row corresponds to one session. When a session is marked with an asterisk, it indicates that it, and the session above it, were recorded in the same mouse.
(PDF)

S2 Table Correlation analysis of classifier weights. (PDF)

S1 Data Classifier codes and data. The zipped folder contains matlab code files (*.m) with classifier codes and matlab data files (*.mat) with the single trial data used for classification. The folder also includes a readme file (*.docx) with a description of the code and the data files.
(ZIP)

S1 Fig Analysis of recorded units’ depth. **A**. schematic of electrode site locations. Shanks are separated 200 μm from each other, while sites within a shank are separated 100 μm. Thus the horizontal span is 600 μm and the vertical span is 700 μm. **B.** An image of an olfactory bulb section with a scale bar imposed. The yellow 2.0 mm bar was generated using the Olympus DPController software (version 1,2,1,108). The red bar was created in Adobe illustrator (scaled according to the yellow bar and rotated). The image shows that along the direction of electrode penetration, the length of the external cell layer is approximately 800 μm. **C.** Recording site locations for the vaginal secretion dataset (set 1). Data from all sessions were combined. In our recordings, we do not precisely measure the medial-lateral aspects, so for this analysis all probes were aligned to the same medial-lateral position. Depth of 0 corresponds to the location at which the first recording sites contact the brain surface. Although consistent across recordings, this measure is also prone to error, explaining why in some cases, units were recorded at extreme superficial or deep positions. Overall, the set of unit positions shows that AOB sampling was expansive and not restricted to the anterior or posterior aspects. **D.** A histogram of recorded unit positions along the depth axis. Positions were pooled across all medial-lateral positions. Colors for single and multi-units in D correspond to those used in C. **E** and **F**, as in C, and D, for the urine dataset (set 2). **G** and **H**, as in C, and D, for the across secretions dataset (set 3).
(EPS)

S2 Fig Fraction of units responding to various secretions. **A.** Fraction of units responding to stimuli within each of the three different dilutions used. To contribute to a given bar, a unit has to respond to at least one of the four stimuli given in that dilution. As expected, the fraction of responding units increases with stimulus concentration. **B.** Histogram showing the number of stimuli eliciting a response in any given unit. The minimum is 1, because by definition, the dataset contains units that respond to at least one stimulus. **C, D:** same as A and B for the urine dataset. **E**,**F**, same as A, and B but for the across secretion dataset. Here, the different bars in E represent different stimulus sources rather than different dilutions.
(EPS)

S3 Fig Detailed description of classifier performance. **A-C.** Pairwise (A), across dilutions (B), and general (C) classification for state discriminations with vaginal secretions. **D-F.** Pairwise (D), across dilutions (E), and general (F) classification for strain discriminations with vaginal secretions. **G-I.** Pairwise (G), across dilutions (H), and general (I) classification for state discriminations with urine. **J-L.** Pairwise (J), across dilutions (K), and general (L) classification for strain discriminations with urine. **M-O.** Pairwise (M), across secretion (N), and general (O) classification for state discriminations with multiple secretions. **P-R.** Pairwise (P), across secretions (Q), and general (R) classification for strain discriminations with multiple secretions. Legends explain the meaning of each line style (same conventions apply to panels A-F and G-L). *best classifier* refers to the best classifier over the 10 repeated training cycles with all units, where the average is over different classifications within a category—e.g. all pairwise strain classifications with urine—hence, it is a single value. *Individual classifiers* refers to the performance of each classifier within a category, averaged over the 10 repeated training cycles. *mean classifier* refers to the average of all the individual classifiers. *best with one unit* refers to the highest classification attained with one individual unit. *individual generalization* refers to the generalization performance obtained with one classifier applied to conditions used to train another classifier. The value represents an average over the 10 repeated training cycles. *mean generalization* refers to the averaged individual generalizations (i.e. across the twelve different comparisons in A,D,G,J,M,P, and the two comparisons in B,E,H,K,N, and Q).
(EPS)

S4 Fig Analysis of generalization across dilutions (stimulus set 1). **A.** Fraction of units responding (blue) and fraction of responses across all units (green) as a function of stimulus dilution. The bars show a population-level monotonic increase in response frequency with concentration. The upper panel shows the same data as in S2A Fig. **B.** Detailed description of specific generalizations, for pairwise classifiers trained with all available units. (For example, 9X3 represents the result of classifying the 9x data by the 3x-trained classifier). The representation does not reveal any consistent relationships between similarity of dilutions and success of generalization. **C.** Reordering of units according to weights assigned by a classifier trained to discriminate strain across three dilutions (for non-estrus females). **D.** Reordering of units according to weights assigned by a classifier trained to discriminate strain for only one dilution (3x dilution, simple pairwise classification). Comparison of the panels C and D indicates that only the classifier in C assigns high weights to units that generalize well across dilutions. Many of the influential units in panel D do not generalize well across dilutions.
(EPS)

S5 Fig Analysis of classification with simultaneously recorded units. The analysis is identical to that used for the other classifications (e.g. Fig 3), but applied to only one session for each dataset. Results represent averages over 3 (rather than 10) repeated training cycles. **A-B**. State and strain classification with vaginal secretions, 24 units (8 single, 16 multi-units). **C-D.** State and strain classification with urine, 15 units (12 single, 3 multi-units). **E-F**. State and strain classification across secretions, 26 units (18 single, 8 multi-units).
(EPS)

S6 Fig Analysis of response profile correlations for the VS dataset(stimulus set 1). **A.** All units in the VS dataset, sorted according to the *absolute* weight of a general strain classifier with all units. **B.** Magnified view of top 20 units shown in A. **C.** Histograms of pairwise correlations between response profiles of units in the dataset. Pairwise correlations were calculated for pairs of rows in the matrix. Recall that each row represents the mean responses of a given unit to each of the twelve stimuli. The top panel shows the histogram of the (unique) pairwise correlations among the top 20 units (190 pairwise correlations). The bottom panel shows the histogram of the pairwise correlations among the remaining units. **D**, **E**, and **F** are like A,B, and C, except that the relevant classifier is for state rather than strain.
(EPS)

S7 Fig Analysis of response profile correlations for the urine dataset (stimulus set 2). Same conventions and panel layout as S6 Fig, applied to dataset 2 (urine).
(EPS)

S8 Fig Analysis of response profile correlations for the multiple secretion dataset. Same conventions and panel layout as S6 Fig, applied to dataset 3 (across secretions).
(EPS)

S9 Fig Principal component analysis of neuronal responses in each of the datasets. Each panel shows the results of principal component analysis of the respective dataset. The top plot in each panel shows the percentage of variance explained by each PC. The other three plots show, for each dataset, the first three Principal components. **A:** VS (set1), **B**: urine (set 2), **C**. across secretions (set 3). The first PC in A can be coarsely associated with stimulus concentration, whereas those in B and C are more reminiscent of the strain, and the secretion type, respectively.
(EPS)

S10 Fig Comparison of perceptron and support vector machine (SVM) classifiers. Each panel shows all individual classifications within a category. Performance is shown for all units in each dataset. SVM performance is shown in blue and perceptron performance is shown in red. Although the two types of classifiers show similar performance in simple discriminations and for a small number of units, with more difficult classifications and particularly when more units are used, SVM classifiers reveal a clear advantage over perceptron classifiers. In these panels, for each discrimination and each number of units, the *best* (rather than the mean) classifier across 10 repeats is shown. **A-B**. State and strain classification with vaginal secretions, 92 units. **C-D.** State and strain classification with urine, 51 units. **E-F**. State and strain classification across secretions, 164 units.
(EPS)

S11 Fig Classification performance with individual units. For each discrimination, classifiers were trained with each individual unit separately. The bars indicate the number of cases with at least 80% (black) or 100% correct (green) classification. Although perfect classifications can often be attained with single units for the simple pairwise discriminations (yellow horizontal bars), this is not the case for the more generalized classifications across all dilutions (orange bars), and even more so, across dilutions and traits (red bars). **A:** urine (stimulus set 1), **B:** vaginal secretions (stimulus set 2), **C:** across secretions (stimulus set 3).
(EPS)

S12 Fig Demonstration of response specificity to undiluted urine samples and to a control stimulus. **A-D:** Examples of four different units validating the use of undiluted (1 fold) urine as a stimulus. **A, B**: Examples of two units (raster displays and traces superimposed) that show selective responses to one 1x urine sample, but not to another. **C.** An example of a unit that does not show rate elevation to one urine concentration, but does show elevation to a 10F dilution of a different urine sample. **D.** A unit that responded to one 1F urine stimulus did not respond to delivery of a 50 mM potassium solution. We note that 50mM is considerably higher than reported values for potassium in mouse urine [49]. Note that even when a 1F dilution is delivered to the nostril, inevitably, the effective dilution that reaches vomeronasal sensory neurons is lower. Thus, non-specific activation of vomeronasal sensory neurons with undiluted urine does not occur in our experiments. **E-F**: Examples of AOB units (not included in the current dataset) that responded to female urine, but did not show firing rate modulations in response to ringer’s solution.
(EPS)
