# Supplementary material for: Extracting Behaviorally Relevant Traits from Natural Stimuli: Benefits of Combinatorial Representations at the Accessory Olfactory Bulb
Source: PLoS Comput Biol. 2016 Mar 3;12(3):e1004798. doi: 10.1371/journal.pcbi.1004798 (PMC4777510; doi:10.1371/journal.pcbi.1004798)
Supplement: S1 Table — Each row corresponds to one session. When a session is marked with an asterisk, it indicates that it, and the session above it, were recorded in the same mouse. (PDF) [file pcbi.1004798.s001.pdf]

Table S1

**dataset**	**session no**	**multi unit**	**Single unit**	**total**
VS	1	2	0	2
VS	2	2	1	3
VS	3	6	5	11
VS*	4	3	4	7
VS	5	16	8	24
VS*	6	6	10	16
VS	7	15	8	23
VS*	8	4	2	6
total				92
urine	1	3	12	15
urine	2	1	4	5
urine*	3	1	0	1
urine	4	5	4	9
urine	5	8	1	9
urine*	6	8	4	12
total				51
UVS	1	6	8	14
UVS*	2	6	14	20
UVS	3	4	4	8
UVS*	4	4	6	10
UVS	5	1	7	8
UVS*	6	2	4	6
UVS	7	5	6	11
UVS*	8	5	7	12
UVS	9	1	5	6
UVS*	10	8	18	26
UVS	11	3	11	14
UVS	12	3	1	4
UVS*	13	4	3	7
UVS	14	7	11	18
total				164
