# Supplementary material for: Extracting Behaviorally Relevant Traits from Natural Stimuli: Benefits of Combinatorial Representations at the Accessory Olfactory Bulb
Source: PLoS Comput Biol. 2016 Mar 3;12(3):e1004798. doi: 10.1371/journal.pcbi.1004798 (PMC4777510; doi:10.1371/journal.pcbi.1004798)
Supplement: S2 Table — (PDF) [file pcbi.1004798.s002.pdf]

Table S2

**dataset**	**Mean+SD of correlation coefficients for state and strain classifier over 10 repeated training cycles**	**Significant correlations among the 10 repeats**	**mean+SD of correlation coefficients for repeated training cycles of the state classifier**	**Significant correlations among the 45 pairwise comparisons**
**VS (set 1)**	0.02±0.02	0	0.99±0.003	45
**urine (set 2)**	-0.09±0.02	0	0.99±0.003	45
**across secretions (set 3)**	0.10±0.04	1*	0.92±0.01	45

*: The one significant correlation has a value of 0.2.
