# Supplementary material for: Extracting Behaviorally Relevant Traits from Natural Stimuli: Benefits of Combinatorial Representations at the Accessory Olfactory Bulb
Source: PLoS Comput Biol. 2016 Mar 3;12(3):e1004798. doi: 10.1371/journal.pcbi.1004798 (PMC4777510; doi:10.1371/journal.pcbi.1004798)
Supplement: S1 Data — The zipped folder contains matlab code files (*.m) with classifier codes and matlab data files (*.mat) with the single trial data used for classification. The folder also includes a readme file (*.docx) with a description of the code and the data files. (ZIP) [file pcbi.1004798.s003.zip › supporting data and code/read me.docx]

The folder contains the data on which classifications were made, and basic analysis functions, from which all other analyses are derived. The code has been annotated to facilitate its understanding. Additional codes and data in a more raw form can be obtained from the corresponding author upon request.

**Data files (mat files)**

The folder contains three data files (mat files), one for each stimulus set:

classmat_set1 (for the vaginal secretion set)

classmat_set2 (for the urine set)

classmat_set3 (for the across-secretion dataset)

Each of these files was created from the individual single trial response data. Within each file, the relevant variables are:

**X**: An m by 5000 array, where m is the number of units included in the dataset, and 5000 is the number of unique trials generated for subsequent classification. Each of the 5000 columns represents the population responses across one trial, where each of the m elements is the single-trial response from one unit.

**T:** A 1x5000 array, where the i’th element indicates the stimulus presented for the i’th response in X. For example, if T(1) is 3, then the first column of X represents single trial responses to the third stimulus. In these datasets, T can assume integer values in the range 1-12 (since there are 12 distinct stimuli in each dataset).

**stim_names:** a cell array with one element for each of the distinct stimuli. The values in T correspond to the corresponding entries in stim_names. Thus, if T(j) = R, then, then j’th column in X, corresponds to responses to the stimulus whose name is stim_names{R}.

**Code (m files)**

The function ***analyze classifications*** implements the core procedure of classification. The function calls the ***train_perceptron*** function which relies on MATLAB functions contained in the neural network toolbox. ***analyze_classifications*** is called with a single argument, (1, 2, or 3) indicating which stimulus set to analyze. The result of the analysis (which can take a long time, especially if many repeats are made) are saved in a file called **result_classmat_setN.mat** where N is the value of the argument with which the function as called (corresponding to the stimulus set).

The function ***analyze_svm_classifications*** applies a support vector machine classifier to the data in one of the three stimulus sets. The stimulus set number is the only input to this function. The function loads the result of the ***analyze_classifications*** and thus can only be called after the latter is run. For example, the call to ***analyze_svm_classifications(2)*** loads the datafile ***result_classmat_set2,*** which is be generated by calling ***analyze classifications(2).*** The ***analyze_svm_classifications*** function requires the **result_classmat_setN** file because it uses the same sequence of units applied in each stage of the perceptron classification. Its output, with the result of the SVM classification, is saved in a file called **SVM_result_classmat_setN** (e.g. **SVM_result_classmat_set2** for the second stimulus set).

Yoram Ben-Shaul

January 2016
